# Turing milk into pro-apoptotic oral nanotherapeutic: *De novo* bionic chiral-peptide supramolecule for cancer targeted and immunological therapy

**DOI:** 10.7150/thno.70568

**Published:** 2022-02-21

**Authors:** Wangxiao He, Zhang Zhang, Wenguang Yang, Xiaoqiang Zheng, Weiming You, Yu Yao, Jin Yan, Wenjia Liu

**Affiliations:** 1 Department of Medical Oncology, The First Affiliated Hospital of Xi'an Jiaotong University, Xi'an 710061, PR. China.; 2 Department of Talent Highland, The First Affiliated Hospital of Xi'an Jiaotong University, Xi'an 710061, PR. China.; 3 Institute for Stem Cell & Regenerative Medicine, The Second Affiliated Hospital of Xi'an Jiaotong University, Xi'an 710004, China; 4 General Surgery Department, Tang Du Hospital, Fourth Military Medical University, 710032 Xi'an, Shaanxi, China; 5 National & Local Joint Engineering Research Center of Biodiagnosis and Biotherapy, The Second Affiliated Hospital of Xi'an Jiaotong University, Xi'an, 710004, PR. China.

**Keywords:** Chirality, Peptide, Biomimetic supramolecule, Oral medication and Cancer therapy

## Abstract

Chirality in biomolecules is ubiquitous in our world, but oral nanomedicines constructed from chiral peptides are extremely rare, principally because of the immature nanofabrication and inadequate bioavailability of chiral nanostructures.

**Methods:** To realize the oral administration of chiral peptides and break through their forbidden zone in intracellular space, a chiral-peptide supramolecular (DPAICP) camouflaging with the membrane from milk-derived extracellular vesicles (ME) was developed herein through an aqueous-based growth method of chiral peptide Au(I) infinite covalent polymer (DPAICP) involving in organothiol D-peptides and Au^3+^, and a feasible camouflage technology using ME.

**Results:** DPAICP@ME possessed favorable pharmaceutical properties to remain stable during the gastrointestinal absorption and blood circulation, and showed the satisfactory tumor accumulation through oral medication. Expectedly, oral DPAICP@ME played its predetermined role *in vivo* to restore p53 signaling pathway for cancer therapy in B16F10 homograft malignant melanoma model, LLC Lewis orthotopic transplantation model of lung cancer and patient-derived orthotopic xenograft (PDOX) mice model of colon cancer. Moreover, oral DPAICP@ME augmented the action of immunotherapy by Anti-PD1 through the further T-cell activation.

**Conclusion:** The *de novo* design of the bionic chiral-peptide supramolecule provides a practicable strategy for the construction of biomimetic chiral peptide-derived nanostructures that can be taken orally, and likely boosts chiral nanomedicine discovery efforts for a wider range of diseases including cancer.

## Introduction

Chirality, or handedness, in molecules is ubiquitous in our world, and its major performance is the appearance of left- and right-handed opticity, so-called enantiomers 1-3. Nevertheless, in the biological world, molecules of life including carbohydrates, nucleic acids and proteins appear basically in only one handedness, which generate the enantioselectivity in biological reactions 1, 2. In dramatic consequences, the chirality of enantiomers contributes directly to their biological and pharmaceutical activities, resulting in different physiological effects 4. In especial, natural peptides are composed of L-enantiomeric amino acids, usually providing binding sites to specifically be recognized only by natural L-proteases, hence the fundamental change in backbone-side-chain connectivity and geometry make D-peptides cannot be recognized by natural L-proteins proteases, thereby dramatically increasing its resistance to proteolysis 5, 6. Inspired by this enantioselectivity, a growing number of D-enantiomeric peptides also termed chiral peptides were developed to overcome the biggest pharmaceutical barrier of natural L-peptides - the susceptibility to proteolysis and the subsequent incredibly short half-life. Profited from the favorable proteolysis resistance, D-enantiomeric peptides have been approved to be capable of regulating the protein-protein interaction (PPI) efficiently, and some of them has been consequently approved for clinical application, including but not limited to the therapy for metabolic diseases, chronic infectious diseases and cancer 7, 8.

While some success has been achieved in the clinical translation of therapeutics derived from D-enantiomeric peptides, significant challenges remain to develop their oral therapeutic agents, thereby limiting their clinical application. Oral delivery has advantages over the current regime of therapy by injection or infusion, especially for chronic disease such as cancer therapy with long-term dosing requirements, because the oral drug can provide a long-time and continuous exposure of target cells in a relatively lower thus safer concentration with continuing-administration in separated time periods, resulting in the enhanced efficacy and depressed side effects 9, 10. Moreover, oral delivery is the choicest route for drug administration due to its non-invasive nature, which presents the advantage of avoiding pain and discomfort associated with injections as well as eliminating contaminations 9, 10. Thus, there is a compelling need to develop the reliable oral dosage forms of D-peptides that inherit their action for PPI regulation.

Fortunately, the favorable proteolysis resistance endows D-peptides with the capacity to withstand the multi-protease environment in digestive tract 11, 12, yielding the precondition for oral administration. Even so, oral dosage is difficult for them, because the absorption of D-enantiomeric peptides from gut into circulation remains a formidable obstacle 13, 14. To address it, extracellular vesicles from cow milk provide a potential feasible solution, which can be absorbed efficiently from the gastrointestinal tract* via* the “neonatal” Fc receptor, FcRn 15. By this way, with successes on the oral delivery of miRNA and small-molecular drugs, cow milk extracellular vesicles (ME) presented enough cross-reactivity to be used as oral delivery vehicles in human patients 16, 17. However, ME delivery suffered from laborious and unmanageable cargo loading, which severely restrict its clinical translation 18. What's worse, it can be expected that the relatively large molecular weight and unfixed hydrophilcity as well as electric properties of peptide would further complicate the peptide loading into ME. Thus, it remains a challenge to efficiently and reliably camouflage chiral peptides with ME.

Besides, for a long time, intracellular space is the forbidden zone for the chiral peptides towards PPI regulation, because of the ineffective cellular internalization of D-peptides 19-21, which had approved by previous study that chiral peptides exclusively enriched in cell envelope other than cytoplasm or nucleus 22. To overcome the barrier of cytomembrane penetration, three general strategies have emerged: 1) microinjecting D-peptides directly into cells 23, 24, 2) conjugating D-peptides with cell penetrating peptides (CPPs) 25, 26 and 3) encapsulating D-peptides with liposome 27. Although effective, microinjection is impractical to be applied *in vivo*, and CPPs conjugation as well as liposome encapsulation always suffered from the fussy manufacturing process and rapid remove by the liver and spleen 28-30. Thus, there is a compelling need to develop new strategies for promoting cellular uptakes and targeting activity of D-peptides, extending its application from extracellular targets to intracellular targets. Nanomedicines have emerged as a promising method in general for facilitating the internalization of therapeutic cargos 31, 32. In this case, kinetics characteristics of the drug can be changed, allowing the preferential cellular uptakes through endocytosis or micropinocytosis 33-35. Thus, turning D-peptides into a well-defined nanomedicine is a feasible method to realize the efficient cellular internalization 12, 36, 37. As for the nanofabrication techniques, some chiral biological nanostructures have been produced by lithography and molecular self-assembly 2, 38, 39, but it is remaining a challenge to reliably fabricate three-dimensional chiral peptide-derived nanostructures on a large scale.

To realize the oral administration of chiral peptides and break through their forbidden zone in intracellular space, we attempted to turn mike into an oral nanotherapeutic by replacing milk proteins with *de novo* supramolecules assembled from a pro-apoptotic chiral peptide termed MVP. For the well-defined nanostructures of chiral-peptide derived supramolecule, we devised an “one-pot”, two-step and aqueous-based growth method involving organothiol D-peptides and Au^3+^ to construct a **D-p**eptide **A**u **i**nfinite **c**ovalent **p**olymer (DPAICP). Next, the ME membrane was separated with milk proteins through ultracentrifugation, and the medicative and absorbable artificial milk can be obtained after camouflaging the chiral-peptide supramolecular assembly with ME membrane. As expected, DPAICP@ME played its predetermined role *in vivo* through oral administration to restore p53 signaling pathway for cancer therapy in B16F10 homograft malignant melanoma model, LLC Lewis *in situ* model of lung cancer and patient-derived orthotopic xenograft (PDOX) mice model of colon carcinoma. Importantly, oral DPAICP@ME augmented the action of immunotherapy by Anti-PD1 through the further T-cell activation. The *de novo* design of the ME membrane camouflaged chiral-peptide supramolecular assembly provides a practicable strategy for the construction of well-defined chiral peptide-derived nanostructures that can be taken orally, and likely boost chiral nanomedicine discovery efforts for a wider range of diseases including cancer.

## Results and Discussion

### The design and construction of DPAICP@ME

**Figure 1:**
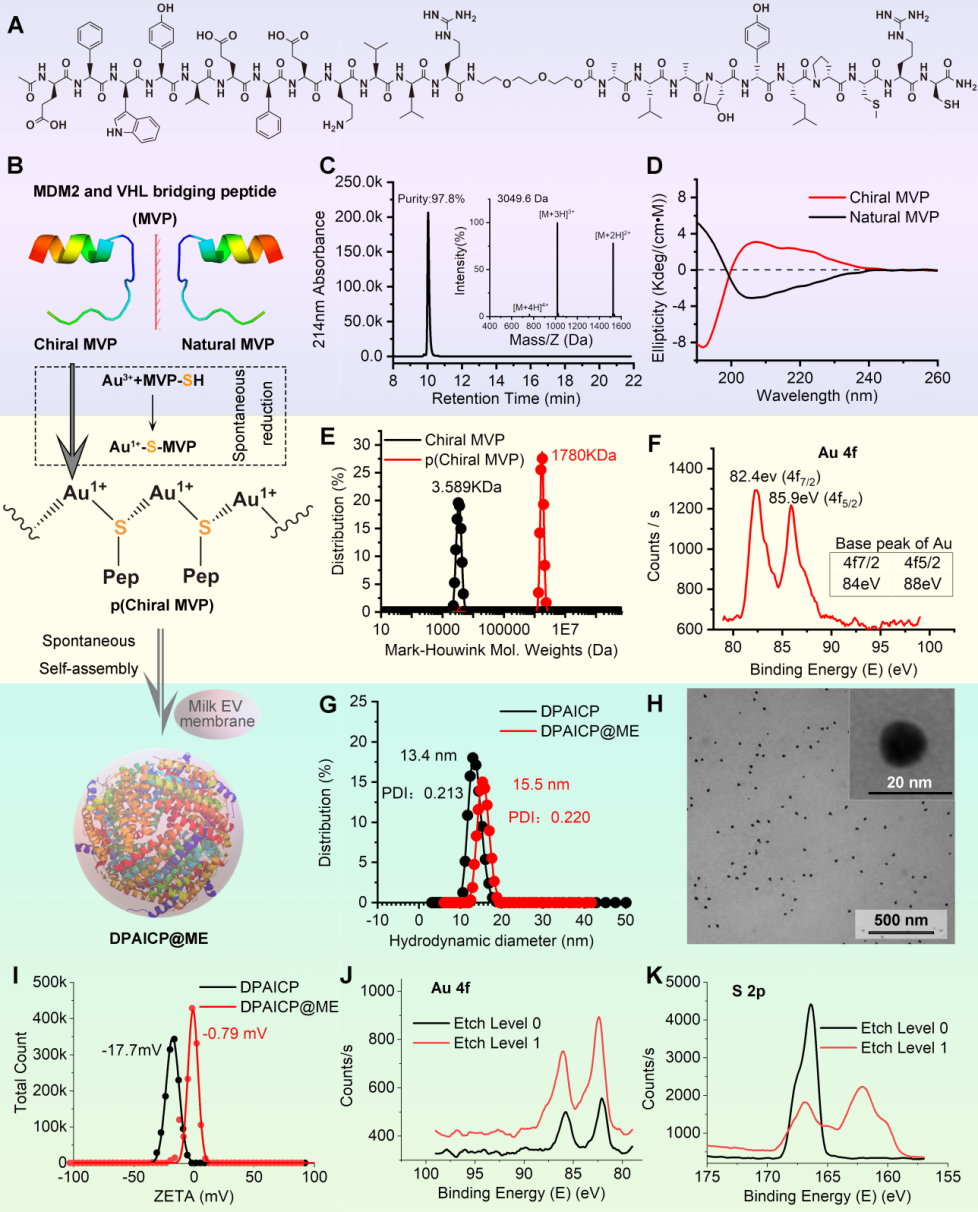
Design, synthesis and characteristics of DPAICP@ME. (A) Molecular structural formula of chiral MVP peptide. (B) Schematic diagram of the DPAICP@ME synthesis. (C) HPLC-MS analysis of the molecular weight and purity of chiral MVP peptide. (D) Circular dichroism spectrum of chiral MVP peptide and natural MVP measured at room temperature in 5mM phosphate buffer including 10% trifluoroethanol. (E) Mark-Houwink molecular weight of chiral MVP and p(chiral MVP). (F) X-ray photoelectron spectroscopy analysis of Au 4f in p(chiral MVP). (G) Hydrodynamic diameters of DPAICP and DPAICP@ME measured in PBS by DLS. (H) TEM image of DPAICP@ME. (I) Zeta potential of DPAICP and DPAICP@ME measured in PBS at room temperature. (J&K) X-ray photoelectron spectroscopy analysis of Au 4f (J) and S 2p (K) at the surface and core of DPAICP@ME.

In this proof-of-concept research, the D-peptide used here was a p53 activator that can specifically degrade MDM2 33 - the most important p53 negative regulation protein in cancer cells 40, 41. As outlined in Figure 1A, the designed chiral peptide termed chiral MVP (MDM2 and VHL bridging peptide) was a protein targeting chimeras consisting of three functional parts: a dextrorotary dodecameric motif binding to MDM2 with high-affinity (^D^MBP) 33, a flexible tripolymer ethylene glycol linker, and a motif binding to an E3 ubiquitin convener called “Von Hippel Lindau factor” (VHL) 42, 43. For the conjugation between Au^3+^ and D-peptide, an extra mercapto-contained chiral Cys residue and a hydrophilous chiral Arg residue were introduced to the C-terminus of chiral MVP. Notably, for comparative study, natural MVP were also designed, in which ^D^MBP was changed to a levorotatory dodecameric motif binding to MDM2 with the almost identical affinity (Figure 1B) 44. The chiral MVP was prepared by standard total chemical synthesis of peptide in FMOC chemistry as our previous reports 45-48, and >95% purified peptide with the correct molecular weight can be obtained in large scales (Figure 1C). Chiral MVP adopted a α-helical conformation as evidenced by characteristic circular dichroism (CD) spectral peaks at 195 nm, 208 and 222 nm (Figure 1D). Meanwhile, the CD spectra of natural MVP is consistent with the known typical α-helical secondary structure, which is symmetrical about the X-axis to the chiral MVP (Figure 1D), illustrating the symmetrical optical rotation between chiral MVP and natural MVP. Additionally, both of the chiral MVP and natural MVP have the approximate affinity at nanomole level to bind MDM2 and MDMX, which were measured by fluorescence polarization analysis as shown in Figure S1.

As outlined in Figure 1B, DPAICP@ME was constructed *via* a one-pot aqueous-based self-assembly including two steps: 1) chiral peptide oligomerization and 2) nanostructures growth with ME membrane fusion. In the first step, the aqueous Au^3+^ was reduced by the thiol in chiral MVP and formed a polymeric structure of [MVP-S-Au(I)]_n_ termed p(Chiral MVP) as evidenced by the increased Mark-Houwink molecular weight (Figure 1E). Furthermore, the X-ray photoelectron spectroscopy (XPS) analysis showed that peaks of Au 4f p(Chiral MVP) in presented at 82.4 eV and 85.9 eV, which is a negative shift from the base peaks of Au 4f in gold elemental (Figure 1F). This XPS result suggested the coordinate bond between Au and thiol, which is in line with the expected chemical construction of p(Chiral MVP) shown in Figure 1B and further proved by the characteristic infrared absorption given by the Au-S bond in fourier transform infrared (FT-IR) spectroscopy (Figure S1). Next, in step 2, the self-assembly of p(Chiral MVP) into a spherical nanostructure would perform spontaneously and be driven by aurophilicity among Au (I) 37. The dynamic light scattering (DLS) analysis and FT-IR spectroscopy confirmed the formation of DPAICP, which has the uniformly distributed hydrodynamic diameter of 13.4 nm (Figure 1G) and characteristic IR absorbance given peptide (Figure S2). Besides, MEs were extracted from fresh milk by ultracentrifugation, and their grain diameters were identified by the TEM image and DLS analysis (Figure S3A-B). In addition, the obtained MEs were further identified by the biomarker of exosome specificity, including CD9, CD63, CD81 and TSG101 (Figure S3C). ME membranes were obtained by the ultrasonication and the succedent ultracentrifugation, and their fracture morphology were characterized by TEM image and DLS analysis (Figure S3D-E). Next, these ultrasonic-broken ME membranes were added into the solution of DPAICP colloid, and the uniform as well as monodispersed DPAICP@ME nanoparticle (Figure 1H) can be obtained after 5-min magnetic stirring. As shown in the TEM image of DPAICP@ME (Figure S4), a low-density shadow layer with the thickness about 2nm can be found around the dense nanoparticle, suggesting the successful ME membrane coating. Additionally, the increased hydrodynamic diameter (Figure 1G) and the negative-shifted ZETA potential (Figure 1I) of DPAICP@ME in comparison with DPAICP further proved this coating. The XPS result of DPAICP@ME further supported this result, in which the signal of Au was not obvious at the surface of DPAICP@ME, but significantly increased after etching with Ar plasma (Figure 1J), suggesting that the Au-free thin film covered on the surface of DPAICP@ME. Moreover, the electron energy band of S 2p measured by XPS showed that the S at the surface of DPAICP@ME is the thiol form in contrast to the coordinate form after etching (DPAICP@ME), in line with the thiol form in the protein of the ME membrane (Figure 1K).

### DPAICP@ME possessed favorable pharmaceutical properties

**Figure 2:**
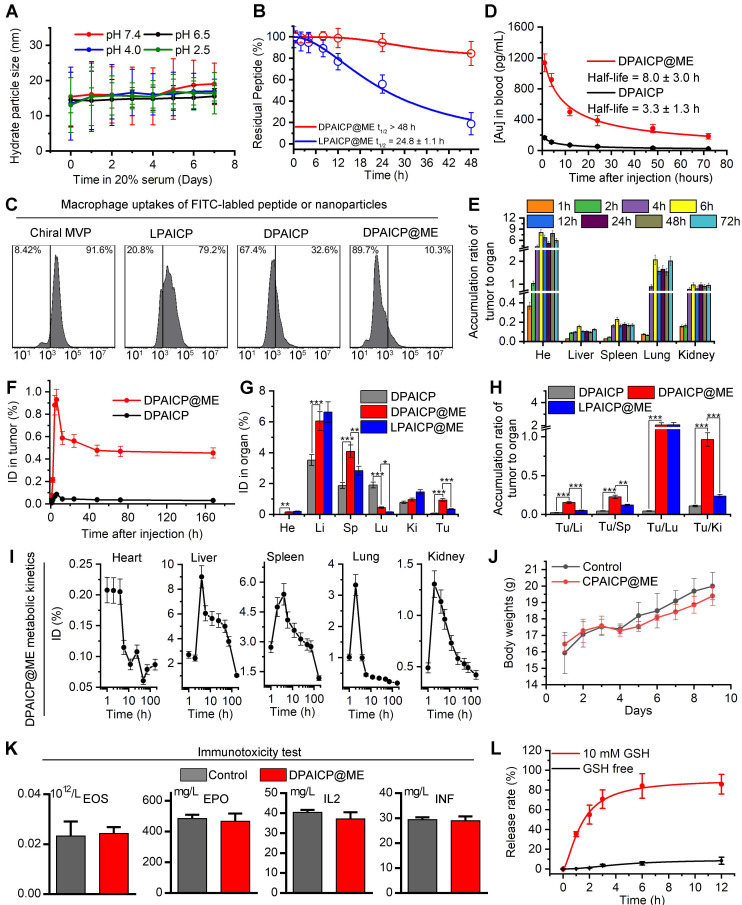
DPAICP@ME possessed favorable pharmaceutical properties to remain stable during the gastrointestinal absorption and blood circulation, and showed the satisfactory tumor accumulation through oral medication. (A) Hydrodynamic diameters of DPAICP@ME measured at pH 2.5, 4.0, 6.5 and 7.4 in PBS containing 20% serum. (B) Proteolytic resistance of DPAICP@ME against 10μg/mL trypsin and 10μg/mL chymotrypsin in standard PBS buffer (pH 6.0) at 37 ℃. (C) Cellular uptake of Cy3-labeled chiral MVP, LPATIC, DPAICP and DPAICP@ME by macrophage cell line: RAW 264.7 cells after the 4 h incubation. (D) Tumor accumulation curves of DPAICP@ME and DPAICP in C57/B6 mice by measuring the concentrations of Au in blood at different time points post injection. The error bars were based on the standard deviations (SD) of triplicate samples. (E) Tumor-to-background (normal organ or tissue) ratios for DPAICP@ME measured different time points after intragastric administration. (F) Time-dependent quantifies of DPAICP@ME or DPAICP in tumor measured by ICP-MS. (G) Biodistribution of DPAICP@ME, DPAICP and LPAICP@ME in C57 mice bearing B16F10 cells ig. dosing. (H) Tumor-to-background (normal organ or tissue) ratios for DPAICP@ME, DPAICP and LPAICP@ME. (I) tissue kinetics of DPAICP@ME in C57/B6 mice after ig. dosing. Serial sacrifices were carried out at 1, 2, 4, 6, 12, 24, 48, 72 and 168 h after dosing. Several organs/tissues, including heart, liver, spleen, lung, and kidney were isolated to determine gold concentrations by ICP-MS. The data were shown as mean ± SD. (J) Body weights of C57/B6 mice after ig. Dosing 20 mg/Kg DPAICP@ME. (K) Immunogenicity of DPAICP@ME in immune-competent C57BL/6 mice (n = 5/group) as measured by the concentration of Eosnophils (EOS), cytokines IL-2 (IL2), IFN-γ (IFN) and erythropoietin (EPO). The data from each group are presented as the mean ± SD. (L) Cargo release curve of DPAICP@ME in GSH or GSH-free PBS buffer, measured by HPLC. Date was presented as the mean ± SD.

After DPAICP@ME construction, we firstly tested its colloidal stability in neutral and acidic pH. As shown in Figure 2A, DPAICP@ME maintained monodispersed during the one-week incubation in PBS buffer at pH 7.4, 6.5, 4.0 and 2.5, suggesting its credible colloidal stability in digestive tract. Besides, to verify the proteolytic resistance of DPAICP@ME against the protease in gastrointestinal tract, 100 μM DPAICP@ME or its L-enantiomer, LPAICP@ME, were incubated with 10 μg/mL trypsin and 10 μg/mL chymotrypsin in standard PBS buffer (pH 6.0) at 37 ℃. Expectedly, after 48-hour incubation, more than 80% L-peptide in LPAICP@ME was degraded, while over 90% D-peptide in DPAICP@ME maintained integrity (Figure 2B). Besides, to explore macrophage uptakes, fluorescein isothiocyanate (FITC)-labeled chiral MVP, LPAICP, DPAICP, and DPAICP@ME were synthesized, and their cellular uptakes by macrophage cell line, RAW264.7, were examined using quantitative flow cytometry and Laser Scanning Confocal Microscopy (LSCM) image. As shown in Figures 2C and S5, D-enantiomerization and ME coating obviously decrease macrophage uptakes. Of note, DPAICP@ME inherited the ability of DPAICP and LDAICP to internalize cancer cells (Figure S6). Theoretically, the suppression of macrophage uptakes and the optimization of stability would endow DPAICP@ME with the prolonged blood circulation. To test this hypothesis, we detected the blood circulation of DPAICP@ME and DPAICP through the quantification of ^197^Au by inductively coupled plasma mass spectrometry (ICP-MS) on healthy C57/B6 mice. Time-dependent ICP-MS measurements of mice blood yielded metabolic kinetics, supporting the prolonged blood-circulation time after ME coating (Figure 2D). Collectively, these results illustrated that ME coating endowed DPAICP@ME with favorable pharmaceutical properties to remain stable during the gastrointestinal absorption and blood circulation.

### Oral DPAICP@ME possessed the satisfactory tumor accumulation, therapeutic safety and the GSH-triggered releases

In theory, the optimized stability and macrophages escape would further promote the tumor accumulation of DPAICP@ME *via* the enhanced permeability and retention (EPR) effect.^49^-^52^ To explore the tumor targeting, B16F10 melanoma homo-grafting mice model were established by subcutaneously injecting 5×10^5^ tumor cells at the right side of the hips, and ICP-MS was employed to quantify the ^197^Au from tissues of tumor-carrying mice after the DPAICP@ME or DPAICP oral administration at the dosage of 2 mg/Kg. As shown in Figure 2E, the time-dependent accumulation ratio of DPAICP@ME in tumor to normal organ or tissue supported the tendency towards tumor accumulation. More importantly, DPAICP@ME showed nearly ten times as many tumor accumulations as ME-free DPAICP (Figure 2F). Besides, although DPAICP@ME, DPAICP and LPAICP@ME presented liver, spleen, kidney and lung accumulation, DPAICP@ME showed the increased tumor accumulation and the decreased healthy tissue accumulation (Figure 2G-H). This result suggested that D-enantiomerization and ME coating have the ability to optimize the tumor targeting, presumably because of enhanced stability and macrophages escape. This viscera accumulation compelled us explore the metabolism of the DPAICP@ME. As shown in Figure 2I, ICP-MS measurements showed over 80% of DPAICP@ME accumulated in the heart, liver, kidney spleen and lung were eliminated within 5 days, indicative of the erasability of DPAICP@ME from organism.

Additionally, we further investigated the acute toxicity of DPAICP@ME after single oral administration towards C57/B6 mice with healthy immune system at a dosage of 20 mg/Kg, which was ten times the therapeutical concentration. As expected, no obvious loss of weight can be found in the one week after administration (Figure 2J), and no hepatotoxicity (Figure S7), nephrotoxicity (Figure S8), cardiotoxicity (Figure S9), and hematotoxicity (Figure S10) was observed. More importantly, high-dose administration of DPAICP@ME had no effect on the four immunogenicity indexes shown in Figure 2K, suggesting the absence of anaphylaxis and T cell immunogenic responses to our engineered exosome. In short, these results supported our design for a tumor-targeting and secure-safe DPAICP@ME for oral administration.

Another key design of DPAICP@ME is its responsiveness to intracellular reduction environment. The release kinetics of chiral MVP was monitored by High-performance liquid chromatography (HPLC) upon the incubation with or without 10 mM GSH that can simulate the intracellular reduction environment 34, 53. As expected, DPAICP@ME generally maintain its integrity with about one-tenth peptide release after a 12-hour incubation without GSH, while incubation with 10 mM GSH resulted in four-fifths cumulative release within 6 hours (Figure 2L). As a result, the released chiral MVP can result in the restoration of p53 action and consequent cycle arrest as well as apoptosis, which was supported by the western immunoblotting analysis (Figure S11), flow apoptosis analysis (Figure S12A-B) and cell cycle analysis (Figure S12C-D) of B16F10 melanoma cells upon the incubation of 1 μM DPAICP@ME.

### Oral DPAICP@ME potently suppressed tumor progression in mouse homo-grafts of B16F10 melanoma

**Figure 3:**
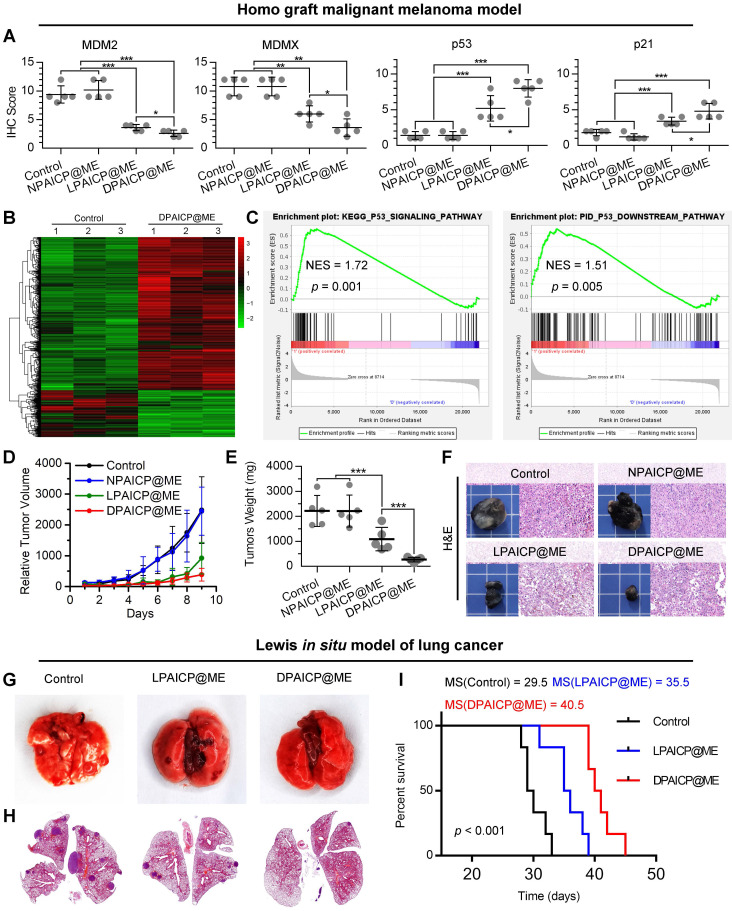
DPAICP@ME potently suppressed tumor progression in mouse homo-grafts of B16F10 melanoma and *in situ* LLC LUAD. (A) Immunohistochemical score of MDM2, MDMX, p53 and p21 in mouse homo-grafts of B16F10 melanoma after indicated treatment. (B) Hierarchical clustering of genes differentially expressed in DPAICP@ME-treated mouse homo-grafts of melanoma compared with mock-treated ones. (C) GSEA analysis between DPAICP@ME treatment and mock treatment involved in the p53 signaling pathway and p53 downstream pathway. (D) Tumor growth curves in mice subcutaneously inoculated with melanoma. Data are presented as mean ± s.e. (n = 5/group). (E) weights of the tumors excised at the end of the experiment. *p* values were calculated by *t*-test (*, *p* < 0.05; **, *p* < 0.01; ***, *p* < 0.001). (F) Representative photos and H&E staining of tumors excised at the end of the experiment. (G and H) Representative photographs (G) and H&E staining (H) of lung in homo-grafts of *in situ* LLC LUAD model with indicated treatments. (I) Survival of mice after injection of LLC cells with the indicated different treatments.

To further verify the *in vivo* performance, we firstly explored the pharmacokinetic characteristics and biodistribution of DPAICP@ME both by oral medication (ig) and intravenous injection (iv). As show in Figure S13A and S13B, both oral medication and intravenous injection resulted in the similar blood circulation time as well as tumor accumulation. Moreover, oral medication and intravenous injection of DPAICP@ME caused in similar therapeutic effects on tumor suppression (Figure S13C) and p53 restoration (Figure S13D). These results suggested that oral medication was consumed as an alternative to intravenous injection for DPAICP@ME therapy. Next, we explored the p53-restoration action of DPAICP@ME in mice bearing B16F10 murine melanoma with the tumor volume range from 70-150 mm^3^ (n = 5/group). After a 9-day intragastric administration (ig) of isopyknic normal saline (NS, Control), DPAICP@ME, LPAICP@ME and a drug-free carrier termed NPAICP@ME at the dosage of 2 mg/Kg every other day, immunohistochemical staining analysis showed that DPAICP@ME substantially more active than its L-enantiomer LPAICP@ME in degrading MDM2 and its homologous protein MDMX and reactivating p53, in sharp contrast to the placebo NS and NPAICP@ME (Figure 3A and S14). Moreover, the signaling pathway analysis by transcriptome sequencing (RNA sequencing) showed DPAICP@ME triggered 894 differential expressed genes (DEGs) in comparison to NS (Figure 3B), and gene set enrichment analysis (GSEA) disclosed that the top up-regulated pathways in DPAICP@ME-treated mouse homo-grafts of melanoma involved p53 signaling pathway and p53 downstream signaling pathway (Figure 3C). As a result, compared to two placebos (NS and NPAICP@ME), DPAICP@ME potently suppressed the growth of mouse homo-grafts of melanoma with more *in vivo* activity that LPAICP@ME in the inhibition of tumor volume and weight (Figure 3D-F).

### Oral DPAICP@ME potently suppressed tumor progression in orthotopic transplantation model of murine lung adenocarcinoma (LUAD)

Moreover, these results were proved again in an orthotopic transplantation model of murine lung adenocarcinoma (LUAD) in which LLC cells (5×10^5^ per mouse) were intravenously inoculated into C57BL/6 mice. After 2-weeks, 18 mice bearing orthotopic LUAD were divided into three group randomly, and administered intragastrically each other day with 2 mg/Kg DPAICP@ME, LPAICP@ME or equivoluminal NS, respectively in a 14-day treatment period commenced three days after tumor challenge. At day 14 post-administration, in comparison to NS and LPAICP@ME, DPAICP@ME remarkably reduced LUAD burden as supported by the number of pulmonary nodule (Figure 3G) as well as the tumor area (Figure 3H). More significantly, DPAICP@ME prolonged the median survival time of mice (MS) to 40.5 days in sharp contrast to the mock-treated mice of 29.5 days, while LPAICP@ME just improved the MS to 35.5 days that was statistically significantly (*p <* 0.001) lower than the DPAICP@ME group (Figure 3I).

### Oral DPAICP@ME potently suppressed tumor progression a patient-derived orthotopic xenograft (PDOX) model of colon cancer

**Figure 4:**
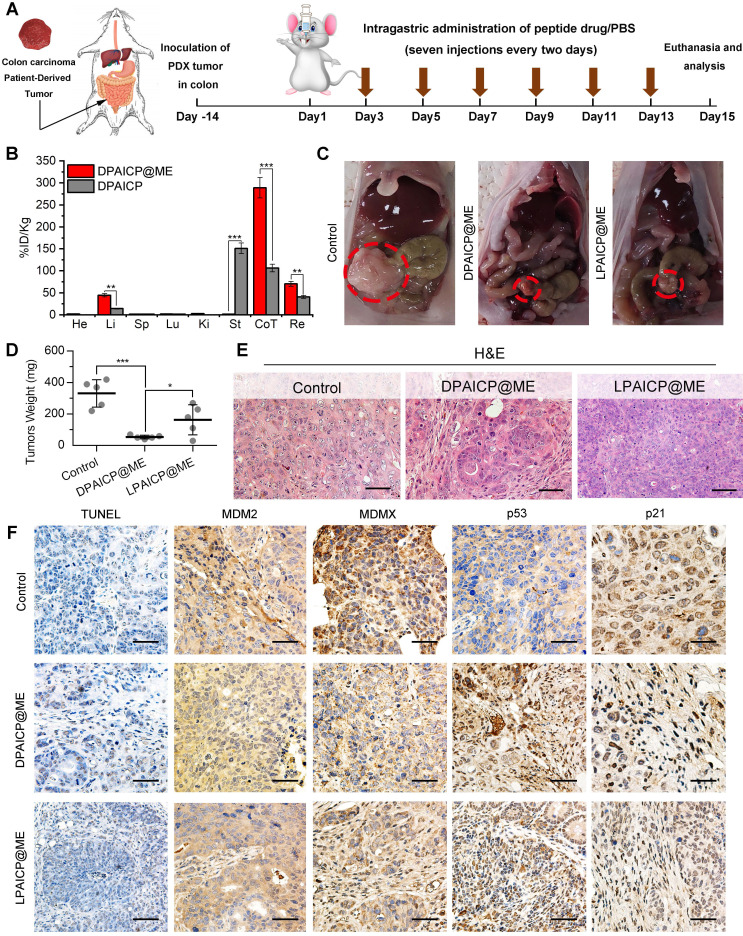
DPAICP@ME suppressed carcinoma progression in a patient-derived orthotopic xenograft (PDOX) model of colon cancer upon oral medication. (A), schematic depiction for model building and treatment. (B) ICP-MS quantification of gold content (from DPAICP@ME or DPAICP) in tumors and organs collected from health PDOX model mice. He, heart; Li, liver; Sp, spleen; Lu, lung; Ki, kidneys; St, stomach; Co, colon; Re, rectum. (C) Photographic images from mice bearing colon carcinoma patient-derived xenograft tumors with the indicated treatments. (D) Photos of the tumors collected from mice with the indicated treatments. (E) Representative photographs of the H&E tissue sections of tumors from mice with the indicated treatments. (scale bar: 60 μm). (F) Representative photographs of the H&E, TUNEL and IHC-stained tissue sections of tumors from mice with the indicated treatments. (scale bar: 60 μm).

Additionally, to further challenge the potency of oral DPAICP@ME, a patient-derived orthotopic xenograft (PDOX) model of colon cancer was established by implanting tumor tissues from a colorectal cancer patient to the colon of NOD/SCID mice orthotopically (Figure 4A). To verify the tumor targeting, 100 μg DPAICP@ME or DPAICP were administered intragastrically into PDOX mice model comparatively (n = 3/group), and followed the biodistribution measurement by ICP-MS. As shown in Figure 4B, at 6 h after administration, DPAICP was mainly distributed in the stomach, whereas DPAICP@ME was concentrated in colon and rectum in which tumor tissues were implanted, suggesting the enhanced gastrointestinal absorption profited from ME coating. This phenomenon further compelled us to study the therapeutic efficacy of DPAICP@ME on the orthotopic tumor of intestine. Before treatment, the whole-exome sequencing of this PDOX tumor revealed a deleterious mutant KRAS and wild-type p53 (Figure S15), indicative of the applicability of DPAICP@ME therapy. Next, 15 NOD/SCID mice bearing this PDOX of colon cancer were randomly divided into two groups (n = 5/group), and performed an intragastric administration every other day for 14 days of NS (control), 2.0 mg/Kg DPAICP@ME or 2.0 mg/Kg LPAICP@ME (Figure 4A). After the treatment, significantly shrunken tumors can be found in the DPAICP@ME-treated mice in comparison to the mice upon mock treatment (Figure 4C-E), while DPAICP@ME showed more anti-tumor action than LPAICP@ME (Figure 4C-D). This result was further supported by the increased number of apoptotic cells in DPAICP@ME-treated tumor measured by H&E and TUNEL staining (Figure 4E-F). Moreover, down-regulated MDM2/MDMX and up-regulated p53/p21 suggested again that the antitumor mechanism of DPAICP@ME was MDM2 or MDMX degradation and subsequent p53 restoration (Figure 4F). Collectively, these results illustrated that DPAICP@ME potently suppressed carcinoma progression upon oral medication.

### Oral DPAICP@ME augmented immunotherapy in mouse homo-grafts of B16F10 melanoma

**Figure 5:**
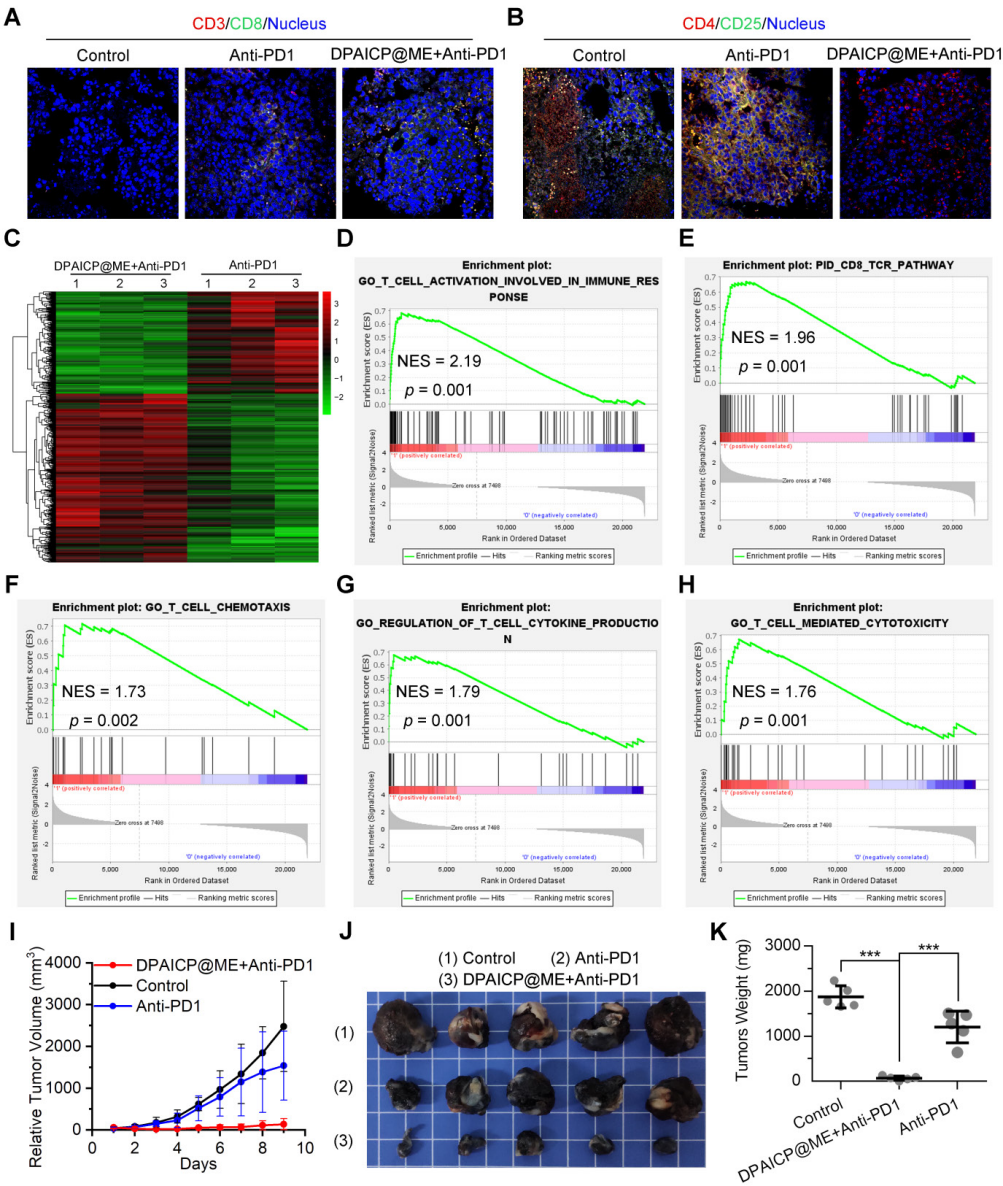
DPAICP@ME augmented immunotherapy in mouse homo-grafts of B16F10 melanoma. (A&B) Immunofluorescence images of cytotoxic T cells (CD3^+^/CD8^+^) (A) and regulatory T cells (CD4^+^/CD25^+^) (B) cells in tumor sections from mice with the indicated treatments (scale bar: 60 μm). (C) Hierarchical clustering of genes differentially expressed in DPAICP@ME/Anti-PD1 combo-treated mouse homo-grafts of melanoma compared with Anti-PD1-treated ones. (D-H) GSEA analysis between Anti-PD1 monotherapy and Anti-PD1/DPAICP@ME combo therapy involved in the T-cell activation in immune response (D), CD8 TCR pathway (E), T cell chemotaxis (F), regulation of T cell cytokine production (G) and T-cell mediated cytotoxicity (H). (I) Tumor growth curves in mice subcutaneously inoculated with melanoma. Data are presented as mean ± s.e. (n = 5/group). (J and K) Photos (J) and weights (K) of the tumors excised at the end of the experiment. *p* values were calculated by *t*-test (*, *p* < 0.05; **, *p* < 0.01; ***, *p* < 0.001).

Besides, as previous report, p53 activation always tended to augment immunotherapy through further T-cell activation.^42^, ^54^ To verify the immunotherapy sensitization of DPAICP@ME, we firstly further analyzed the T-cell related signaling pathway in the RNA sequencing results of mouse homo-grafts of B16F10 melanoma. As expected, GSEA analysis showed marked up-regulated signaling enrichment in CD4^+^ cell, CD8^+^ cell and T lymphocyte after the DPAICP@ME treatment (Figure S16A-C), which was supported again by the increased infiltration of cytotoxicity T cells (CTCs) in DPAICP@ME-treated mouse homo-grafts of B16F10 melanoma (Figure S16D). For further verification, 15 mice bearing B16F10 murine melanoma with the tumor volume range from 70-150 mm^3^ were equally divided into three groups: NS, Anti-PD1 and Anti-PD1/DPAICP@ME combo. Of note, DPAICP@ME was administrated intragastrically as above therapeutic regimen, and the Anti-PD1 -the neutralizing monoclonal antibody against murine PD1- was administrated intravenously at the day 1 and day 5 of the treatment. After the 9-day treatment, compared to Anti-PD1 monotherapy, Anti-PD1/DPAICP@ME combo therapy showed the increased number of CTCs (Figure 5A), the enhanced CTCs activity (Figure S17), and the decreased number of regulatory T-cells (Figure 5B), suggesting the augmented immunotherapy action. This result was supported again by the contrastive GSEA analysis between Anti-PD1 monotherapy and Anti-PD1/DPAICP@ME combo therapy, in which the top-regulated combo-treated tumor included genes involved in the up-regulated T-cell activation in immune response, CD8 TCR pathway, T cell chemotaxis, regulation of T cell cytokine production and T-cell mediated cytotoxicity (Figure 5C-H). As a result, Anti-PD1/DPAICP@ME combo was substantially more active *in vivo* than Anti-PD1 monotherapy in suppressing the tumor progression as evidenced by tumor volume curve (Figure 5I), tumor photo (Figure 5J) and tumor weights (Figure 5K). Taken together, these results illustrate that DPAICP@ME sensitized the anti-tumor action of immune checkpoint inhibitor by hyperactivating the function of CTCs.

## Conclusions

Hereby, to fabricate well-defined chiral peptide-derived nanostructures and realize its oral administration, an extra-small bionic chiral-peptide supramolecular assembly termed DPAICP@ME was designed as oral therapeutic. In details, we devised an “one-pot”, two-step and aqueous-based growth method involving organothiol D-peptides and Au^3+^ to construct a chiral peptide Au infinite covalent polymer (DPAICP) as the content of the engineered exosome, and developed a feasible nanostructures packaging technique using the membrane from cow milk exosomes as the shell of the engineered exosome. In this proof-of-concept research, a D-peptide-derived p53 activator that can specifically degrade MDM2 in cancer cells was used here to construct the DPAICP@ME. Expectedly, DPAICP@ME played its predetermined role *in vivo* through oral administration to restore p53 signaling pathway for cancer therapy in B16F10 homograft malignant melanoma model, LLC Lewis *in situ* model of lung cancer and PDOX mice model of colon cancer. Importantly, oral DPAICP@ME augmented the action of immunotherapy by Anti-PD1 through the further T-cell activation. Thus, we can expect that the combination therapy between DPAICP@ME and immunotherapy has the potential to achieve the eradication in early tumors and partially sensitive advanced tumors with systemic metastasis. In short, the *de novo* design of the ME membrane camouflaged chiral-peptide supramolecular assembly provides a practicable strategy for the construction of biomimetic chiral peptide-derived nanostructures that can be taken orally, and likely boost chiral nanomedicine discovery efforts for a wider range of diseases including cancer. More importantly, while our nanoengineering strategy of biomimetic chiral nanostructures construction helps overcome existing pharmacological obstacles for oral peptide, this chiral chemistry itself is not restricted to nanoengineer peptide *per se* as it is amenable to construct other biomimetic chiral nanostructures derived from proteins, nucleic acids, and other classes of chiral drug compounds as well.

## Supplementary Material

Supplementary experimental section and figures.
