# Supplementary material for: Turing milk into pro-apoptotic oral nanotherapeutic: De novo bionic chiral-peptide supramolecule for cancer targeted and immunological therapy
Source: Theranostics. 2022 Feb 21;12(5):2322–34. doi: 10.7150/thno.70568 (PMC8899570; doi:10.7150/thno.70568)
Supplement: Supplementary file 1 — Supplementary experimental section and figures. [file thnov12p2322s1.pdf]

Supplementary Materials

Turing milk into pro-apoptotic oral nanotherapeutic: De novo bionic chiral-peptide supramolecule for cancer targeted and immunological therapy

Wangxiao He[1,2,3,*,†], Zhang Zhang[4,†], Wenguang Yang[1,2], Xiaoqiang Zheng[3], Weiming You[5], Yu Yao[1], Jin Yan[5,*] and Wenjia Liu[3,*]

[1]Department of Medical Oncology, The First Affiliated Hospital of Xi'an Jiaotong University, Xi'an 710061, PR. China.

[2]Department of Talent Highland, The First Affiliated Hospital of Xi'an Jiaotong University, Xi'an 710061, PR. China.

[3]Institute for Stem Cell & Regenerative Medicine, The Second Affiliated Hospital of Xi'an Jiaotong University, Xi'an 710004, China

[4]General Surgery Department, Tang Du Hospital, Fourth Military Medical University, 710032 Xi'an, Shaanxi, China

[5]National & Local Joint Engineering Research Center of Biodiagnosis and Biotherapy, The Second Affiliated Hospital of Xi'an Jiaotong University, Xi'an, 710004, PR. China.

[†] These authors contributed equally.

[*] Corresponding authors:

Email: hewangxiao5366@xjtu.edu.cn (W. He)

Email: yanjin19920602@xjtu.edu.cn (J. Yan)

Email: wenjialiu@xjtu.edu.cn (W. Liu).

1. Experimental Section

General remarks

All synthetic peptide sources were obtained from China Peptides Co.,Ltd. All other chemicals used in this study were purchased from Sigma-Aldrich unless otherwise specified. $HAuCl_4·XH_2O$ was purchased from Aladdin Chemicals. All products were used as received without further purification.

Synthesis of chiral MVP and natural MVP peptide

All peptides were synthesized on appropriate resins on an CS bio 336X automated peptide synthesizer using the optimized HBTU activation/DIEA in situ neutralization protocol developed by an HBTU/HOBT protocol for Fmoc-chemistry SPPS. After cleavage and deprotection in a reagent cocktail containing 88% TFA, 5% phenol, 5% H2O and 2%TIPS, crude products were precipitated with cold ether and purified to homogeneity by preparative C18 reversed phase HPLC. The molecular masses were ascertained by electrospray ionization mass spectrometry (ESI-MS). Of note, chiral MVP was synthesized by Fmoc-protected D-amino acids, and natural MVP was synthesized by Fmoc-protected L-amino acids.

Synthesis of CPAICP

First, 2 mg of MVP peptide was completely dissolved in a solution containing 500μL ethanol and 1.25mL ddH_20. After that, an aqueous solution of tetrachloroauric acid (HAuCl4·XH2O, 1 mL, 10 mM) was mixed with 500μL NH_2-PEG-SH (MW: 2000, 4mg/ml in deionized water) and 2.25 ml HEPES (100mM, pH 7.0). Then it mixed with the prepared solution containing 2.25 ml deionized water and 2.25ml HEPES (100mM pH 7.0), sonicate for 10 min.

Finally, removed the excess reactants by dialysis tubing (cutoff, 10 KDa) and washed twice by distilled water.

Milk extracellular vesicle isolation.

Milk Extracellular vesicles were isolated from milk by differential centrifugation. was done as described previously (Zhou Q, Li M, Wang X, et al. International journal of biological sciences, 2012, 8(1): 118). Briefly, milk aliquots (15 mL) were added to 25 mL PBS, centrifuged at 3000 rcf for 15 min at 4 °C to remove the fat and the cellular fraction of the milk. The supernatant was further centrifuged at 12,000g for 30 min at 4 °C. The resulting supernatant was filtered through 0.45 mm filter to obtain a clear whey fraction. A 500μl of ExoQuickTM extracellular vesicle precipitation solution (System Biosciences, CA, USA) was added to the 1 ml of filtrate and mixed properly by inverting. The mixture was then allowed to incubate overnight at 4 °C, and it was further centrifuged at 1500g for 30 min to pellet the extracellular vesicles. The supernatant was discarded and the extracellular vesicle pellets were resuspended in 100μl of PBS. The resuspended extracellular vesicles were stored at -20°C for further analyses.

Fabrication of CPAICP@ME

The prepared extracellular vesicles were dissolved in PBS following 10 min ultrasonication at 30 W. Next, CPAICP (volume ratio of extracellular vesicles: CPAICP =1: 200) was added and ultrasonicated at 10 W, 5 s, stopping 2 s for 2 min and then allowed to stand on ice for 20 min. The mixture was stored at 4°C in PBS for further analyses.

Physicochemical properties of CPAICP@ME and its intergradations

The morphology and lattice structure were observed on transmission electron microscopy (TEM), which was performed on a Talos F200X. One portion of the pellet was placed onto a

carbon-coated copper grid for imaging with high-resolution transmission electron microscopy (TEM) and selected area electron diffraction (SAED). The hydrodynamic size distribution (1 mg/mL in PBS, 1 mL) was obtained from the dynamic light scattering (DLS) measurement (Malvern Zetasizer Nano ZS system). For Zeta potential measurement, the nanoparticles (1 mg/mL, 1 mL) were incubated with PBS at different pH at 37 ºC for 30 min, and measured by dynamic light scattering (DLS). The surface chemical structure of modified nanocrystals was evaluated by Fourier transform infrared (FT-IR) spectroscopy (Nicolet 6700) and UV−vis absorption spectra (Shimadzu 3000 spectrophotometer). XPS measurement was performed using an electron spectrometer (Thermo Scientific, ESCALAB Xi+) equipped in a chamber with the base pressure of ca. 2×10^{-8} Torr. The X-rays from the Mg KR line at 1253.6 eV (15 kV, 20 mA) were used for excitation. The specimen was prepared by dropcasting the methanol dispersion of a sample onto a polished copper plate. Photoelectrons were collected in the constant analyzer energy mode with a pass energy of 20 eV. The binding energies were corrected by referencing the binding energies of Au(4f) and S(2p) arising from the substrate.

GSH-responded drug release

To test GSH-responded drug release, CPAICP@ME were dissolved in PBS buffer (pH 7.4) containing 10 mM or no glutathione (GSH), and the nanoparticles were then removed by 14000 g centrifuge. Following this, the supernatants were quantified by HPLC and authenticated by ESI-MASS. Separations were performed at a flow rate of 1 mL/min with a gradient from 5 to 65% of B in 30 min (eluent A: 0.1% TFA/H2O, eluent B: 0.1% TFA in CH_3CN).

Cell culture and viability analysis

B16F10 cell and LLC carrying wild-type p53 was purchased by ATCC, and maintained in

RPMI1640 medium with 10% FBS. All cells were maintained at 37°C in an atmosphere of 5% CO_2.

Apoptosis and cell cycle analysis

Necrosis/apoptosis was evaluated by flow cytometry using the FITC Annexin V Apoptosis Detection Kit (BD Biosciences). Briefly, cells were treated with 1μM drug for 48 h. Cells were then harvested, washed twice with cold PBS, and resuspended in 1×binding buffer at a concentration of 1×10^6 cells/ml. One hundred microliters of the solution (1×10^5 cells) was transferred to a 5-ml culture tube, followed by addition of 5 μl of FITC Annexin V and 5 μl of PI. After gentle vortexing and a 15-min incubation in the dark at room temperature, 400 μl of 1× binding buffer was added to the tube, and cells were analyzed by FACS.

Cells were first serum starved for 12 h, and then treated with drug for 6 h. Next, cells were harvested, washed twice in PBS, and fixed in 70% ethanol on ice for at least 30 min. After that, cells were stained with propidium iodide (PI) solution (50 μg/ml PI, 50 μg/ml RNase A, 0.1% Triton-X, 0.1mM EDTA). Cell cycle distributions were then analyzed based on DNA contents by a flow cytometer (BD Biosciences, NJ).

Western Blot Analysis

The protein fraction of cell lysates was resolved by 10%SDS/PAGE before membrane transfer. Primary antibodies were from Santa Cruz Biotechnology (p53), Proteintech (MDM2, MDMX, p21, GAPDH); secondary antibodies conjugated with horseradish peroxidase were from Calbiochem. MG-132 was purchased from Aladdin. Cycloheximide (CHX) was purchased from MeChemExpress.

RNA-sequencing (RNA-seq) and analysis

RNA was isolated from mouse homo-grafts of B16F10 melanoma after indicated treatments using the Direct-zol RNA MiniPrep Kit (Zymo Research). RNA sequencing libraries were constructed using the NEBNext® Ultra RNA Library Prep Kit for Illumina® (NEB England BioLabs). Fragmented and randomly primed 2 × 150 bp paired-end libraries were sequenced using Illumina HiSeq X Ten. Heat maps and Gene Expression Enrichment Analysis were generated using the Qlucore Omics Explorer 3.2. Pathway analysis was performed using Ingenuity Pathway Analysis (IPA) software.

Toxicity studies

To assess acute toxicities of oral CPAICP@ME, we monitored body weight of all mice over the course of treatment and measured hematological indexes as well as organ function indexes after 10-day treatment. Control mice were only implanted with xenograft tumor, but did not receive any treatment. Forty-eight hours after the final infusion, mice were anesthetized, and blood was collected for complete blood count (CBC) determinations, including a white blood cell (WBC) count with differential, a red blood cell (RBC) count, haemoglobin and a platelet count. Besides, blood serum was collected, and alanine aminotransferase (ALT), aspartate transaminase (AST), blood urea nitrogen (BUN) and creatinine (CRE) were measured by using quantitative enzyme-linked immunosorbent assay (ELISA) kits according to the manufacturer's instructions. Animals were then euthanized with carbon dioxide to retrieve organs, which were washed with deionized water before fixation in 4% paraformaldehyde. The tissues were processed routinely, and sections were stained with haematoxilin and eosin (H&E).

Animal ethics

ALL mice were purchased from the Laboratory Animal Center of Xi'an Jiaotong University. Animals were housed under standard specific pathogen-free conditions with standard chow and typical light/dark cycles. All experimental procedures involving animals were conducted in accordance with Institution Guidelines and were approved by the Laboratory Animal Center of Xi'an Jiaotong University.

Mouse homo-grafts of B16F10 melanoma

Cancer cells were harvested when they reached near confluence by incubation with 0.05% trypsin-EDTA. Cells were pelleted by centrifugation and resuspended in sterile PBS. B16F10 cells (4×10^6 cells/site) were implanted subcutaneously into hip of four- to five-weeks-old male athymic nude mice. When the tumors reached average volume of ~ 50 mm^3, the mice were randomly divided into different groups (five mice per group), and treatment was initiated. Tumor length and width were measured with calipers, and tumor volume was calculated using the following equation: tumor volume (V) = length × $width^2/2$. For histological examination, the tumor, liver, kidney, heart, spleen and lung tissues were fixed with formaldehyde, dehydrated, sliced into 5.0 μm sections and subjected to H&E or immunohistochemical staining. Besides, blood routine examinations (white blood cells, red blood cells and thrombocyte) were carried out at the Clinical Laboratory, the First Affiliated Hospital of Xi'an Jiaotong University, according to the standard clinical laboratory procedures.

Quantification of nanoparticle accumulation and pharmacokinetics using ICP-MS.

The gold content inside any organ can be measured using ICP-MS. WERI-Rb1 tumors or organs were resected, weighed, and placed in 50 ml Falcon tubes. They were then digested with 2 ml of nitric acid and 0.5 ml of hydrochloric acid at 70–80 °C overnight. The tissues appeared digested

and dissolved. The samples were diluted to 50 ml with deionized water and then filtered with 0.22 μm PES filters (Millipore) using a 10 ml syringe. The filtered digest was then processed using ICP-MS and analyzed using a standard curve derived from stock with a known quantity of gold.

In vivo orthotopic lung cancer model

LLC cells (1×10^6 cells per 200 μl) were transplanted into C57BL/6 mice between 6 and 8 weeks old by intravenous injection. After 3 days, the mice were randomly divided into different groups, following three successive treatments every other days with.

Colon-cancer-patient-derived orthotopic xenograft tumor.

At the time of primary tumor reductive surgery, a specimen was cut into about 5 mm pieces and implanted into the subserosa of the cecum of NOD/SCID mice aged 4~5 weeks. After 14 days, mice were administrated intraperitoneally at a dosage of 2 mg/kg every other day. Xenografts were collected for formalin-fixed-paraffin embedding (FFPE), snap frozen in liquid nitrogen or subsequently implanted into another set of mice using the same procedure. The hearts, livers, spleens, lungs, kidneys, tumors were sliced for further H&E, TUNEL and immunohistochemical staining.

Immunohistochemical (IHC) staining

Sections were cut at 5 μm thickness, deparaffinized and rehydrated. Endogenous peroxidase activity was blocked with hydrogen peroxide/methanol, and antigen retrieval was performed in a pH 9.0 TE (Tris-EDTA) buffer by autoclave for 10 min. The resultant tissue sections were then incubated with primary antibodies against p53, MDM2, p21, MDMX and Ki67 at 4°C overnight. After incubation with labeled streptavidin-biotin (LSAB) complex for 15 min, the slides were

stained and visualized by using the iView DAB detection system (ZSGB-BIO, P.R. China). Each stained section was evaluated by a minimum of 10 randomly selected ×20 high-power fields for further statistical analysis.

Immunofluorescence staining

For tissues immunofluorescence, tumor sections were stained with primary antibodies then with the corresponding Alexa Fluor-488 or Alexa Fluor-568-conjugated secondary antibody. Nextly, all slides stained with antibodies were mounted with Vectashield Mounting Medium containing 4′-6-diamidino-2-phenylindole (DAPI). The primary antibodies were used:CD3, CD8(Bioss, China;1:50), CD4(Santa Cruz, USA;1:50), CD25(abcam, USA;1:100). Images were obtained by confocal laser-scanning microscopy (OLYMPUS).

Statistics

Analysis for two groups were calculated using an unpaired two-tailed Student's t-test; comparisons of more than two groups were calculated using a one-way analysis of variance (ANOVA) with Tukey post-analysis, or log-rank test where necessary. Survival analyses and curves were performed and generated according to the Kaplan–Meier method.

2. Supplemental Figure

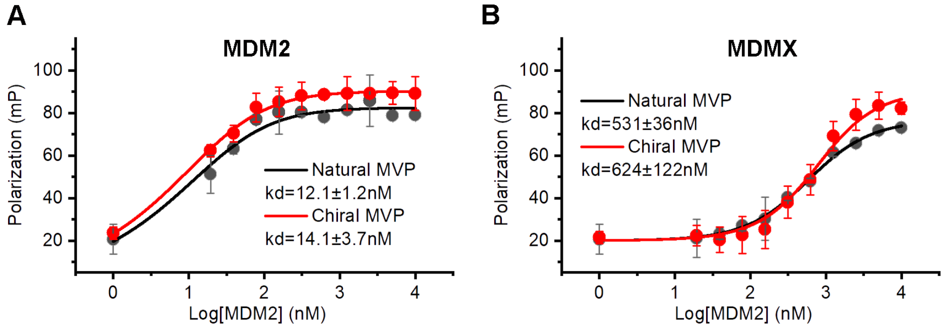

Figure S1. Interaction between natural(L)/chiral(D) MVP to MDM2 (MDMX) characterized by fluorescence polarization

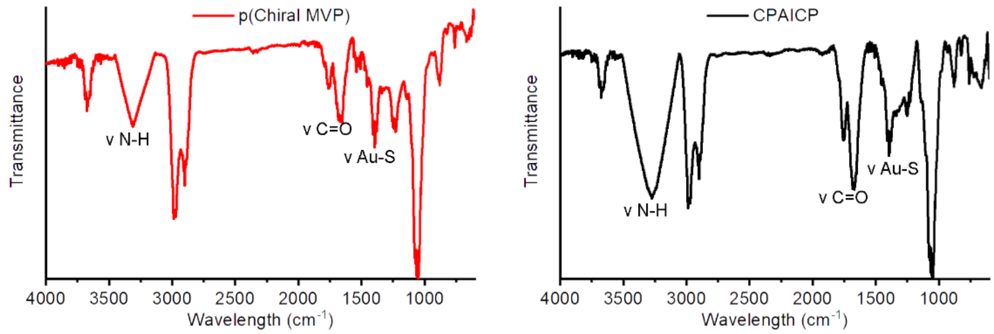

Figure S2. Fourier transform infrared (FT-IR) spectroscopy of p(Chiral MVP) and CPAICP.

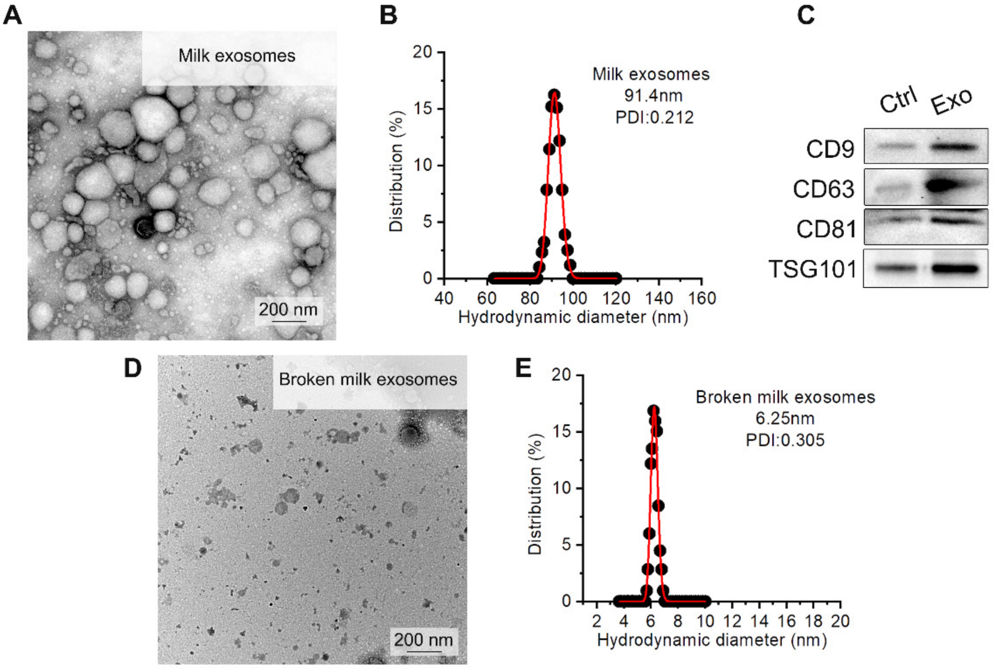

Figure S3. Characterization of milk extracellular vesicles. (A) TEM image of milk extracellular vesicles. (B) Hydrodynamic diameter of milk extracellular vesicles. (C) WB analysis of milk extracellular vesicles. (D) TEM image of broken milk extracellular vesicles. (E) Hydrodynamic diameter of broken milk extracellular vesicles.

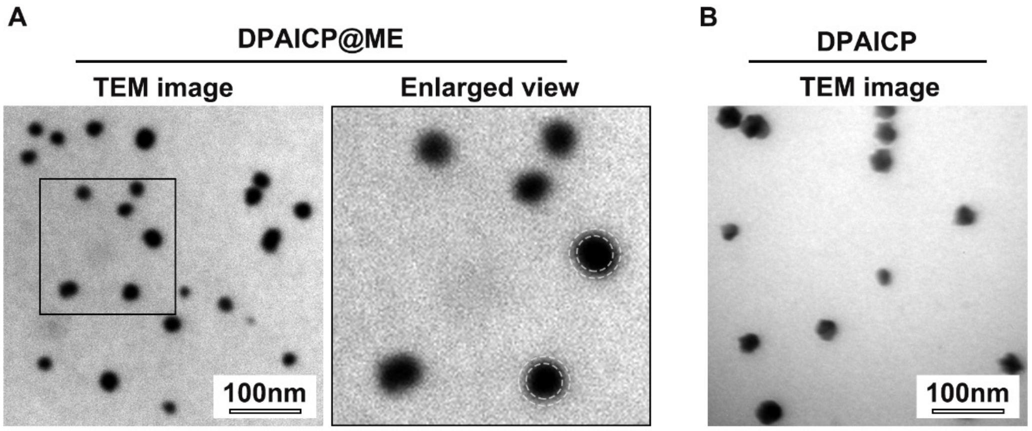

Figure S4. TEM image of DPAICP and DPAICP@ME.

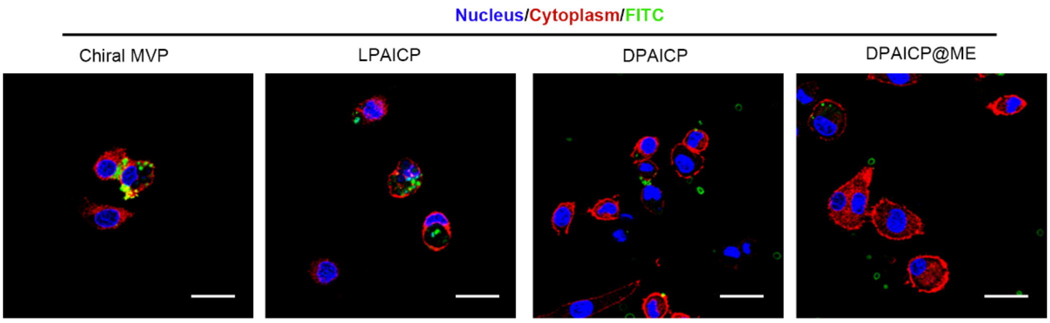

Figure S5. Laser Scanning Confocal Microscopy image of macrophage after incubating with FITC-labeled Chiral MVP, LPAICP, DPAICP and DPAICP@ME

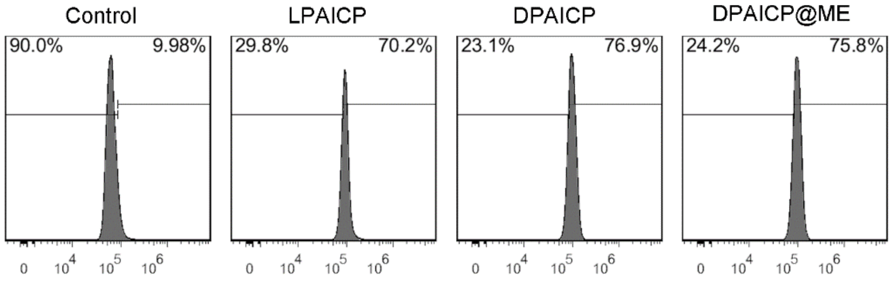

Figure S6. Cellular uptakes of FITC-labeled LPAICP, DPAICP and DPAICP@ME into B16F10 cancer cells.

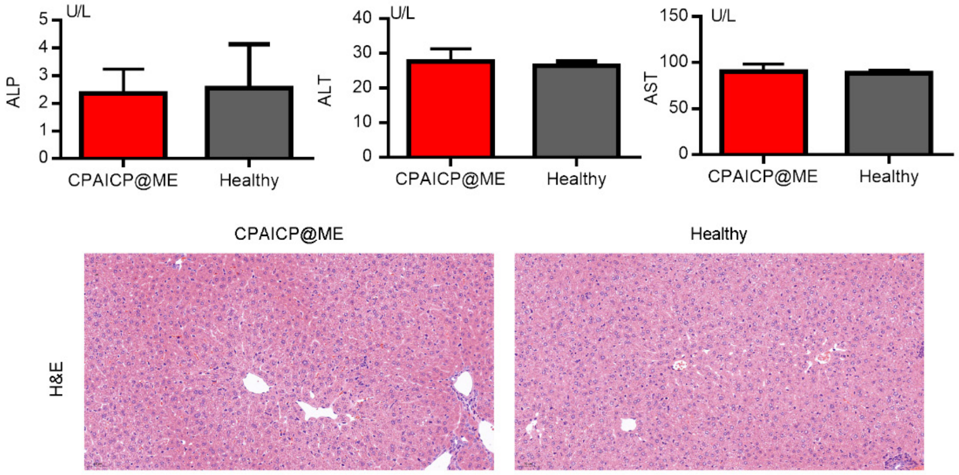

Figure S7. Hepatotoxicity measured by ALP (A Lkaline Phosphatase), ALT (glutamic-pyruvic transaminase), AST (glutamic oxalacetic transaminase) and pathological section of liver.

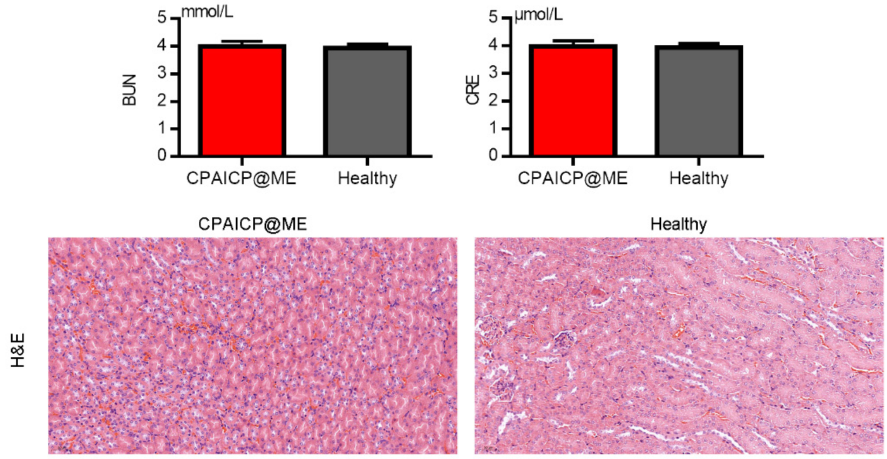

Figure S8. Nephrotoxicity measured by BUN (Blood Urea Nitrogen), CRE (creatinine) and pathological section of kidney.

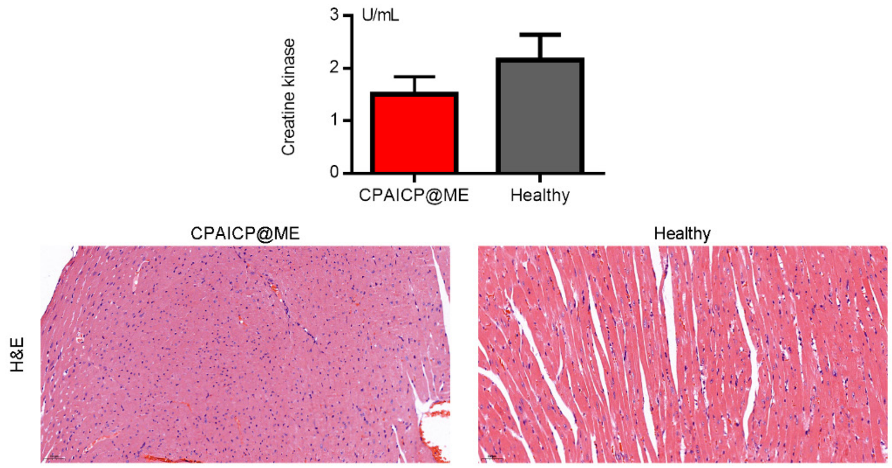

Figure S9. Cardiotoxicity measured by creatine kinase and pathological section of heart.

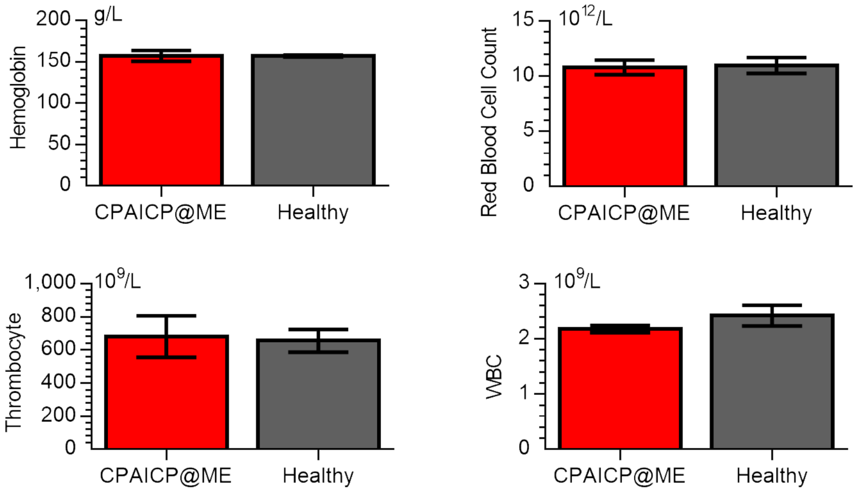

Figure S10. Hematotoxicity measured by hemoglobin, red blood cell count, thrombocyte count and white blood cell count.

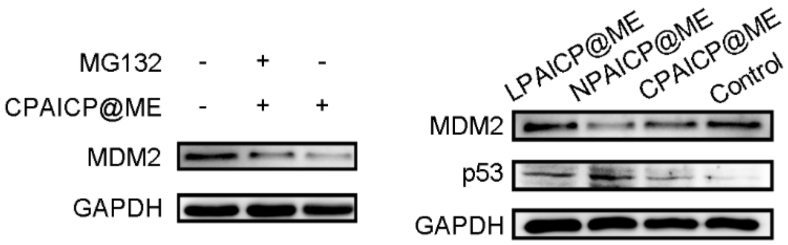

Figure S11. The change in protein expression of MDM2 in B16F10 cells with the 48 h treatments of non-targeting control, CAPAICP@ME, CAPAICP@ME plus MG132. Western blot analysis to monitor the change in protein expression of p53, MDM2 in B16F10 cells with the 48 h indicated treatments.

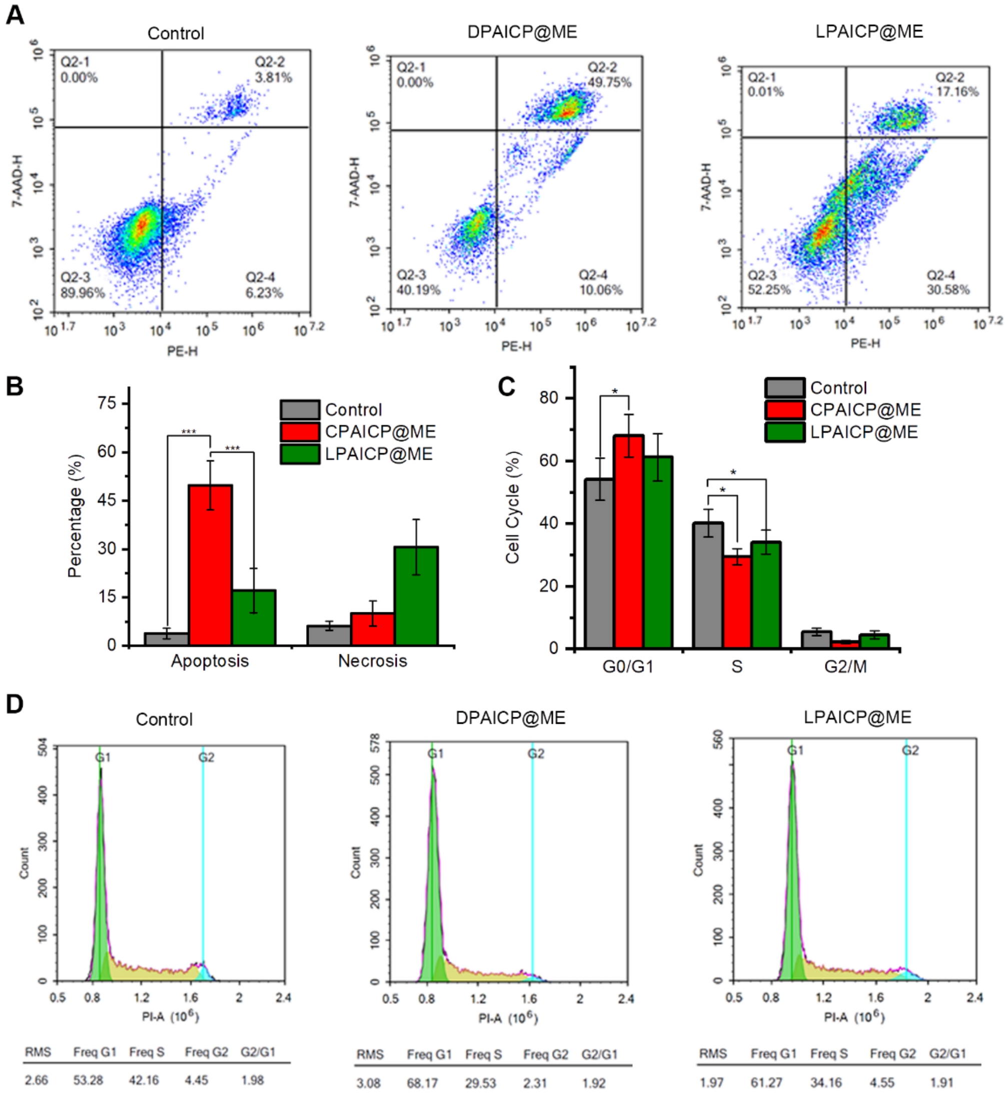

Figure S12. (A&B) Apoptosis analysis of B16F10 cells with different treatments for 12 h. (C&D) Cell cycle distributions of B16F10 cells with different treatments for 12 h. Data were shown as mean ± s.d. p values were calculated by t-test (**, p <0.01; ***, p <0.001).

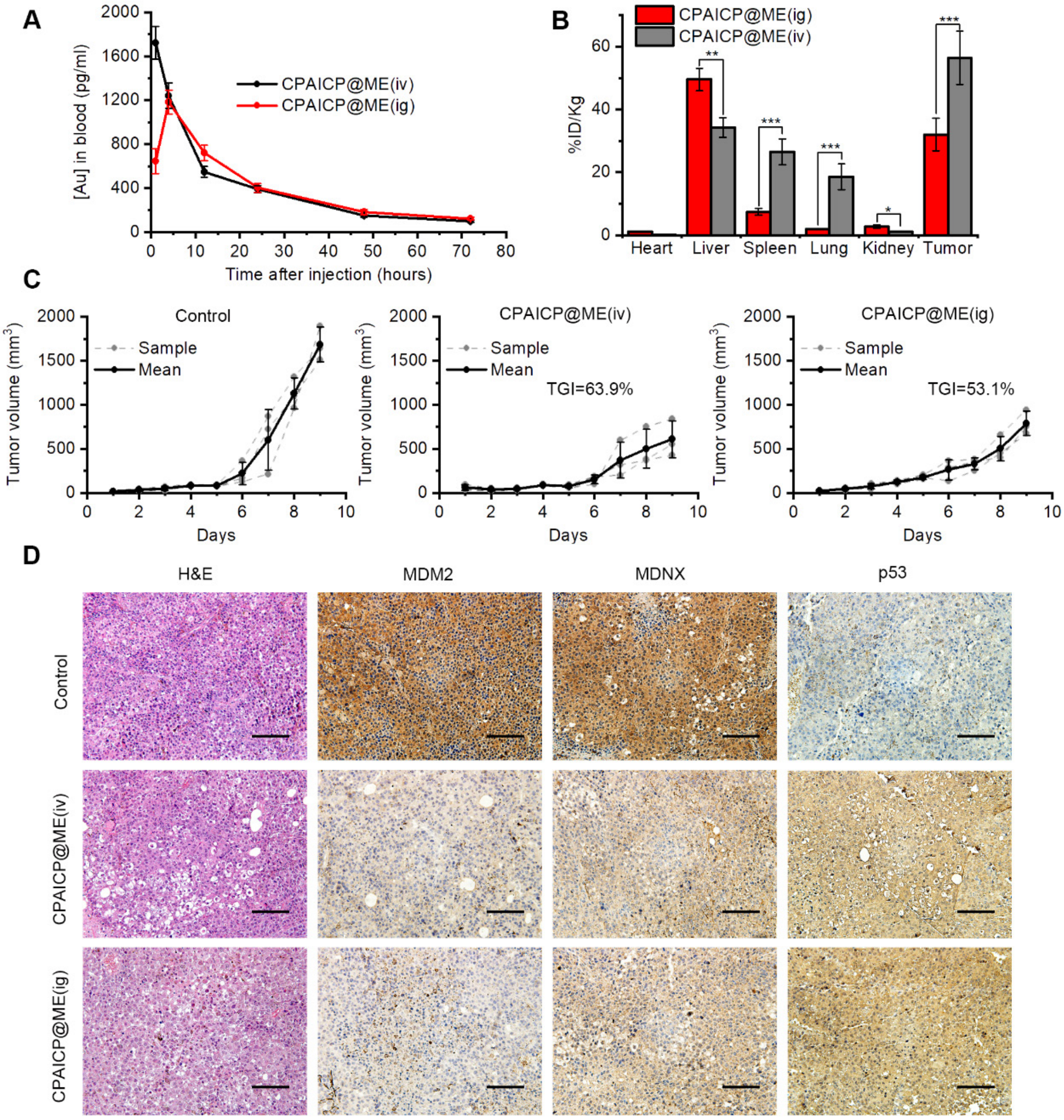

Figure S13. Pharmacokinetic characteristics, biodistribution and Tumor inhibition in mice with DPAICP@ME treatment upon intragastric administration or intravenous injection. (A) Blood-circulation curves in C57/B6 mice by measuring the concentrations of Au in blood at different time points post injection. The error bars were based on the standard deviations (SD) of triplicate samples. (B) Biodistribution of DPAICP@ME in C57/B6 mice upon intragastric administration or intravenous injection. The date was measured at 4th hour after administration and by ICP-MS. (C) Tumor growth curves in mice subcutaneously inoculated with melanoma. Data are presented as mean ± s.e. (n =3/group). (D) H&E image and immunohistochemical staining of MDM2, MDMX, and p53 in mouse homo-grafts of B16F10 melanoma after indicated treatment.

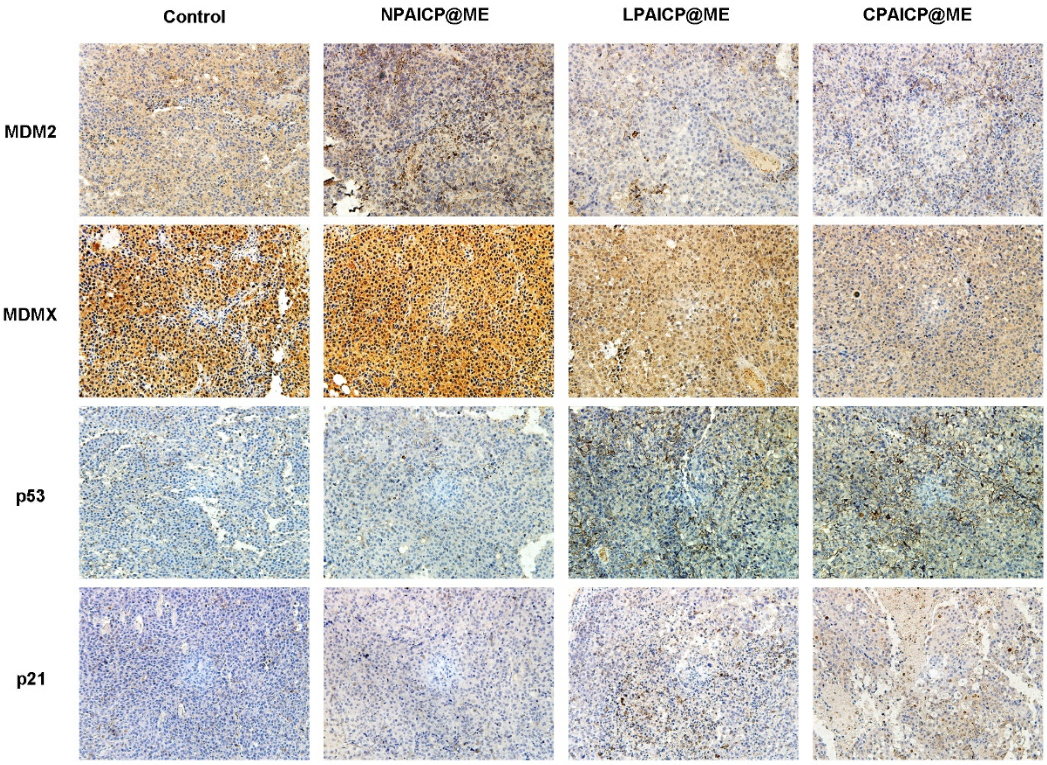

Figure S14. Immunohistochemical staining of MDM2, MDMX, p53 and p21 in mouse homo-grafts of B16F10 melanoma after indicated treatment.

Gene	chromosome	mutation position	base mutations	Residue mutations
TP53	No mutation			
TP73	No mutation			
PTEN	No mutation			
NRAS	No mutation			
BRAF	No mutation			
PIK3CA	3	178916809	T-A	S66T
APC	5	112176756	T-A	V1822D
SMAD4	No mutation			
EGFR	7	55229255	G-A	R521K
AKT1	No mutation			
KRAS	12	25398284	C-T	G12D

Figure S15. Exome screening for common mutations in the PDOX model of pancreatic carcinoma.

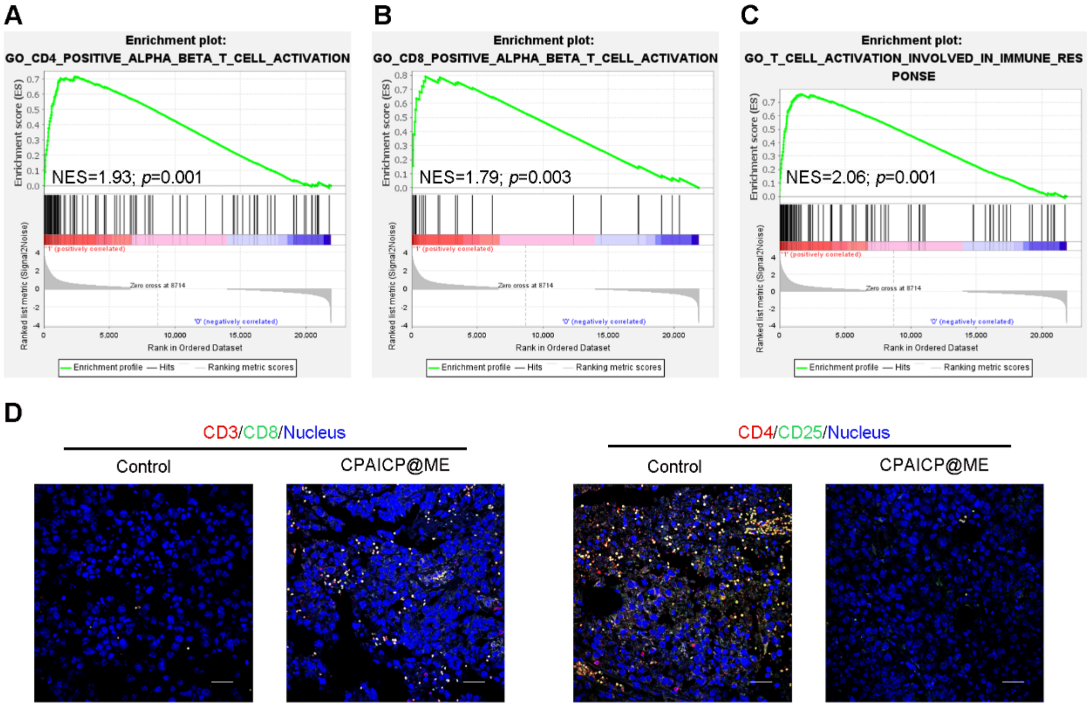

Figure S16. (A-C) GSEA analysis between CPAICP@ME treatment and mock treatment involved in in CD4+ cell (A), CD8+ cell (B) and T lymphocyte (C). (D) Immunofluorescence images of cytotoxic T cells (CD3+/CD8+) and regulatory T cells (CD4+/CD25+) cells in tumor sections from mice with the indicated treatments (scale bar: 60 µm).

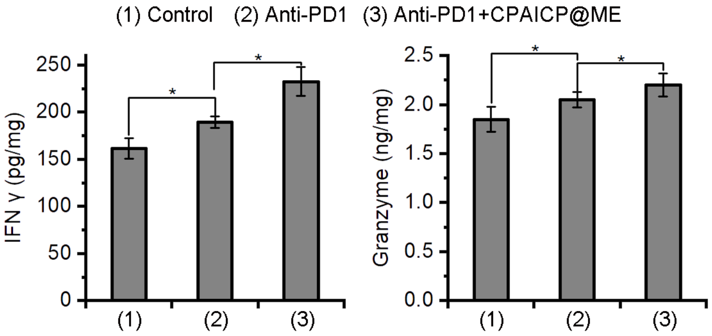

Figure S17. IFN-γ and Granzyme content in tumor sites from mice with the indicated treatments.
